# Tetanus Control in the United States and Global Disaster Settings: Public Health Disparities and Prevention Strategies

**DOI:** 10.3390/medicina62020338

**Published:** 2026-02-07

**Authors:** Olivia Stala, Suhana Patel, Christian Donlon, Syed Shehroz Hussain, Rahim Hirani, Mill Etienne

**Affiliations:** 1 School of Medicine, New York Medical College, Valhalla, NY 10595, USA; 2 Graduate School of Biomedical Sciences, New York Medical College, Valhalla, NY 10595, USA; 3 Department of Neurology, New York Medical College, 40 Sunshine Cottage Road, Valhalla, NY 10595, USA

**Keywords:** tetanus, global health, vaccination coverage, natural disasters, health disparities

## Abstract

Tetanus, a disease caused by the neurotoxin-producing bacteria *Clostridium tetani* (*C. tetani*), remains a serious threat, particularly among individuals who are unvaccinated or under-vaccinated. Although public health guidelines in the United States continue to recommend a well-established, multi-dose vaccination schedule to prevent tetanus, recent revisions to the Centers for Disease Control and Prevention webpage language on vaccine safety prompted renewed public discussion. Despite this, extensive evidence continues to demonstrate the effectiveness and safety of tetanus immunization, and certain demographic groups remain disproportionately at risk. Globally and within the United States, natural disaster zones remain especially high-risk environments for tetanus infection. This review examines the pathophysiology of tetanus, current vaccination recommendations, and the social and geographic inequities that influence vaccine uptake. It also evaluates strategies of protection and prevention. Particular emphasis is placed on tetanus risk in disaster settings, where disrupted infrastructure, greater likelihood of contaminated wounds, and preexisting disparities in vaccination coverage compound vulnerability. A clearer understanding of these factors is essential for strengthening public health preparedness and ensuring equitable protection against tetanus, especially for populations disproportionately affected by disasters.

## 1. Introduction

Tetanus is a potentially fatal, yet fully preventable disease caused by the neurotoxin produced by *C. tetani*, an obligate anaerobic bacterium whose spores are ubiquitous in soil, dust, and animal feces [1,2,3]. Following entry into the body through contaminated wounds, these spores germinate under anaerobic conditions, releasing tetanospasmin, a potent neurotoxin that disrupts inhibitory neurotransmission in the central nervous system [2,3,4,5]. This disruption leads to the hallmark features of tetanus—progressive muscle rigidity, painful spasms, and autonomic instability [2,3,6]. Without timely prophylaxis or advanced supportive care, generalized tetanus can rapidly progress to respiratory failure and death [1,3,7].

Globally, the introduction and expansion of tetanus toxoid–containing vaccines have dramatically reduced the burden of disease [8,9,10]. Reported neonatal tetanus deaths have declined by more than 80% since 2000, and the majority of countries have achieved or are nearing maternal and neonatal tetanus elimination (MNTE). In the United States, routine childhood immunization with diphtheria-tetanus-acellular pertussis (DTaP) vaccines and decennial adult boosters have made tetanus exceedingly rare, with only a few dozen cases reported annually [11,12,13]. Nevertheless, isolated cases and small outbreaks continue to occur, primarily among unvaccinated individuals, older adults, and immigrants with incomplete vaccination histories [14,15,16,17].

Despite clear and longstanding vaccination guidelines, coverage in the United States remains suboptimal in several demographic groups [18,19,20]. Socioeconomic and geographic disparities, healthcare access barriers, underinsurance, and vaccine hesitancy all contribute to uneven immunity [18,19,21,22,23]. Similar inequalities dictate global coverage, evidenced by the difficulty in achieving MNTE in LMIC (low- and middle-income countries) [24]. These inequities, both globally and within the United States, are further magnified in the aftermath of natural disasters, where injuries from debris, population displacement, and disrupted health infrastructure create conditions favorable for *C. tetani* exposure [7,25,26,27,28]. Historical outbreaks following earthquakes, floods, and tsunamis underscore the continued need for vigilance even in well-vaccinated populations [7,27,28,29,30,31,32,33].

This review adopts a dual geographic perspective, integrating both United States–specific guidance and global public health recommendations. U.S.-based guidelines are discussed where clinical practice and emergency response protocols are relevant to domestic care delivery, while World Health Organization (WHO) recommendations are incorporated to contextualize tetanus prevention and control in global and disaster-prone settings. This approach reflects the transnational nature of natural disasters, humanitarian response, and population displacement.

This review provides a comprehensive examination of tetanus pathophysiology, current vaccination recommendations, and existing disparities in vaccine coverage both in the U.S. and globally. Particular attention is given to prevention strategies and outbreak control, with an emphasis on challenges unique to disaster settings. By integrating current epidemiologic data with public health and clinical evidence, this paper aims to highlight gaps in population protection, evaluate effectiveness of existing interventions, and propose strategies to strengthen routine immunization efforts as well as emergency tetanus preparedness and response.

## 2. Methods

### 2.1. Literature Identification and Review Approach

This article is a narrative review intended to synthesize clinical, epidemiologic, and public health literature on tetanus prevention, vaccination disparities, and disaster-related risk. Literature was identified through targeted searches of PubMed/MEDLINE, WHO and CDC databases, and reference lists of key articles. Searches focused on English-language publications from approximately 2000 to 2025 to reflect contemporary vaccination practices, disaster medicine frameworks, and epidemiologic trends.

Gray literature, including public health surveillance reports, policy documents, and guidance from organizations such as the CDC, WHO, and UNICEF, was included where peer-reviewed data were limited or where official recommendations were required to accurately reflect current practice. Formal inclusion and exclusion criteria were not applied, as this review was designed to provide a broad, integrative overview rather than a systematic or quantitative synthesis.

### 2.2. Investigative Focus

The focus of this narrative review can be described using the Population–Concept–Context (PCC) framework. The population of interest includes individuals at risk for tetanus across the lifespan, with particular attention to under-immunized populations. The central concept is tetanus prevention, including vaccination strategies, disparities in coverage, and post-exposure prophylaxis. The context encompasses both routine public health settings and disaster or humanitarian environments globally and within the United States.

## 3. Pathophysiology

Tetanus toxin (TeNT) is produced by the *C. tetani*, an obligate anaerobe capable of forming dormant spores. These spores are often found in soil and environmental debris and typically enter the body through inoculation of an open wound. Once inoculated into anaerobic tissue, spores may remain dormant for days to weeks before germinating and releasing TeNT [1,2,3]. TeNT is a 150 kDa polypeptide composed of a heavy (HC) and light (LC) chain linked by a disulfide bond, with each subunit contributing distinct functional properties with specific binding affinities dictating their interaction within the nervous system [2].

**Figure 1:**
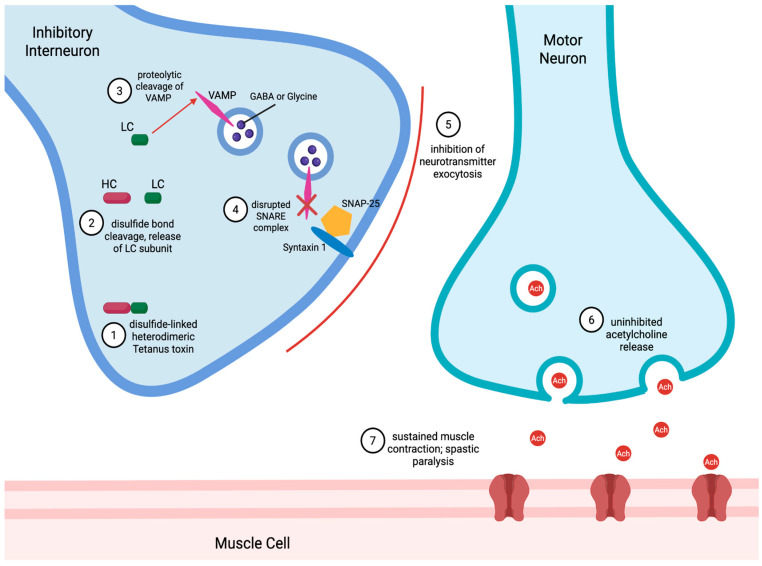
Mechanism of tetanus neurotoxin action. 1. *C. tetani* produces tetanus neurotoxin as a disulfide-linked heterodimeric composed of a heavy chain (HC) and a light chain (LC). 2. Reduction in the interchain disulfide bond releases the LC. 3. The LC, a zinc-dependent endopeptidase, proteolytically cleaves vesicle-associated membrane protein (VAMP, also known as synaptobrevin). 4. This cleavage disrupts the SNARE complex (composed of VAMP, syntaxin, and synaptosomal-associated protein, 25 kDa (SNAP-25)). 5. The disrupted SNARE complex blocks the exocytosis of inhibitory neurotransmitters γ-aminobutyric acid (GABA) and glycine. 6. Loss of inhibitory neurotransmission leads to unchecked acetylcholine release at the neuromuscular junction. 7. Unchecked acetylcholine release results in sustained muscle contraction and spastic paralysis.

The neurotoxin binds motor and sensory neurons in the peripheral nervous system and travels retrograde along the axon towards the spinal cord. Ultimately, the neurotoxin reaches its target: an inhibitory interneuron secreting glycine or γ-aminobutyric acid (GABA) to regulate motor neuron activity. Within the inhibitory interneuron, the disulfide bond is reduced, activating the LC. The LC, a zinc-dependent metalloprotease, cleaves vesicle-associated membrane protein (VAMP)/synaptobrevin, a key soluble N-ethylmaleimide-sensitive factor attachment protein receptor (SNARE) protein required for synaptic vesicle fusion (Figure 1) [2]. This blockade of inhibitory neurotransmitter release results in unchecked excitatory signaling and the characteristic muscle rigidity and spasms of tetanus seen clinically. The HC contributes to both neurotoxicity (via its N-terminal domain) and neuronal specificity (via its C-terminal domain), guiding the toxin to its synaptic targets [4,5].

The tetanus neurotoxin structure is central to its mechanism of action and specificity for inhibitory interneurons. Experimental work has demonstrated that the full-length toxin is required for optimal neuronal binding. In one study, researchers examined binding specificity by injecting mice with various heavy fragments of tetanus toxin and found the full-length protein is more efficient in binding than fragments. Thus, although there are specific domains within the protein structure with higher affinity for binding the neuron, the complete heterodimeric structure is important for functionality [34].

The route and extent of toxin movement along neural pathways directly influence clinical presentation. In localized tetanus, muscle rigidity tends to remain near the site of inoculation. A specific subtype, cephalic tetanus, typically follows cranial or facial wounds and reflects the neurotoxin’s strong affinity for cranial nerve terminals. A reported case of a young patient who sustained scalp abrasions after an earthquake illustrated this predilection: the clinical course began with a cranial nerve VII palsy, followed by involvement of cranial nerves V and VI [35]. Cephalic tetanus most often presents with trismus, facial opisthotonus, and risus sardonicus appearing first. As the disease progresses, the localized symptoms may become more generalized tetanus, characterized by stiffness of the axial musculature, limb involvement, and in severe cases, respiratory muscle spasm leading to respiratory compromise [6].

A unique therapeutic interaction between tetanus toxin and botulinum toxin—produced by *Clostridium botulinum*—has been explored because the two neurotoxins exert opposing physiologic effects. Whereas tetanus toxin blocks inhibitory neurotransmission in the central nervous system, botulinum toxin inhibits acetylcholine release at the neuromuscular junction, reducing peripheral muscle contraction. Given these contrasting mechanisms, investigators hypothesized that botulinum toxin might counteract or attenuate tetanus-induced muscle spasms. In one study, early administration of botulinum toxin during the disease course was associated with reduced severity of symptoms, suggesting a potential adjunctive role in modulating spasticity and preventing progression to more generalized tetanus [6].

## 4. Current Vaccination Guidelines

Vaccination remains the cornerstone of tetanus prevention in both routine public health settings and emergency response contexts. In the U.S., the Centers for Disease Control and Prevention (CDC), through the Advisory Committee on Immunization Practices (ACIP), provides comprehensive guidelines covering immunization across the lifespan—including childhood vaccination, adult boosters, wound management, pregnancy, and special circumstances [36]. Globally, the WHO issues parallel recommendations, which historically have aligned with US guidelines in many areas but differed in booster timing, dosing intervals, and maternal–neonatal implementation strategies [37].

In the United States, ACIP guidelines organize tetanus vaccination across the life course to ensure completion of the primary childhood series, administration of an adolescent booster, and maintenance of protective immunity in adulthood through periodic boosters, with additional provisions for pregnancy, wound prophylaxis, and individuals with incomplete or uncertain immunization histories [12,36,38]. The recommendations allow interchangeability of Td and Tdap for routine boosters, catch-up schedules, and wound management, supporting flexibility in clinical implementation while maintaining consistent coverage against tetanus [12]. Special populations, including pregnant individuals, healthcare personnel, and displaced or undocumented persons, are addressed through tailored dosing strategies intended to rapidly establish or sustain protective immunity [38,39,40,41] A consolidated summary of CDC- and ACIP-recommended vaccination schedules and prophylaxis guidance by population group is provided in Table 1.

The CDC provides explicit guidance for wound management and post-exposure prophylaxis (PEP), emphasizing thorough wound cleaning and debridement, assessment of vaccination history, and administration of TTCV and tetanus immune globulin (TIG) when indicated [40]. For contaminated or major wounds, a booster dose is recommended if more than five years have passed since the last tetanus vaccine, while for clean minor wounds the interval extends to ten years [40]. These protocols have direct relevance in disaster medicine and mass-casualty situations, where traumatic, contaminated wounds are prevalent and immunization histories are often unknown or incomplete.

The CDC recommends Tdap vaccination during every pregnancy, ideally between 27 and 36 weeks of gestation, regardless of prior vaccination history [41]. Individuals with uncertain or incomplete histories should complete a three-dose primary series at zero, four weeks, and six to 12 months, with one of those doses being Tdap [41]. The WHO’s MNTE strategy complements these guidelines by promoting TTCV administration to women of reproductive age, promoting clean delivery and cord-care practices, and implementing supplementary immunization campaigns to maintain community-level protection [8,42].

The CDC also highlights the need for flexible strategies in individuals with uncertain or incomplete records, such as refugees, migrants, and other displaced persons, who often lack formal documentation [42,43]. In these cases, catch-up immunization is prioritized rather than restarting the full series, and a single Tdap dose serves as the initial vaccine for anyone aged ≥ seven years, followed by boosters with Td or Tdap at recommended intervals [42]. Such provisions are critical for ensuring equitable vaccine delivery in emergency, humanitarian, and post-disaster settings.

Globally, the WHO recommends a primary TTCV series beginning at six weeks of age, followed by boosters at 12–23 months, 4–7 years, and 9–15 years [37]. Unlike the CDC guidance, WHO policy does not include universal adult boosters, focusing instead on maintaining immunity through childhood and adolescence. Comparative epidemiologic studies -such as analyses of France, which maintains decennial adult boosters, and the United Kingdom, which does not maintain decennial adult boosters, have shown similar national tetanus incidence rates, suggesting that frequent adult boosters confer limited additional benefit in populations with strong childhood immunization programs [44]. Modeling analyses likewise indicate that protective antibody levels persist for decades after completion of childhood immunization series, and that lengthening booster intervals is a cost-effective alternative [45].

Recent evidence also supports coadministration of Tdap with other vaccines, including influenza, human papillomavirus (HPV), and coronavirus disease of 2019 (COVID-19) vaccines, without reducing immune response or increasing adverse events [46]. Coadministration is permitted under CDC immunization guidance and has been shown to improve adult vaccination coverage, which may facilitate efficient delivery when multiple vaccines are administered during large-scale immunization efforts [46,47].

The COVID-19 pandemic provides a contemporary example of how large-scale systemic disruptions can undermine routine vaccination, including TTCV. Deferred clinical visits, reduced access to preventive care, and overburdened health systems led to significant declines in immunization coverage worldwide [23]. The pandemic demonstrated how large-scale systemic disruptions can undermine vaccine continuity and increase susceptibility to preventable diseases such as tetanus.

Across existing guidelines, there is broad consensus that lifelong protection against tetanus requires completion of the primary vaccine series and periodic booster doses, with special attention to wound prophylaxis and maternal immunization. The United States emphasizes decennial adult boosters and wound-specific vaccination, whereas WHO recommendations prioritize child and adolescent immunization alongside maternal–neonatal protection in resource-limited regions. Evidence questioning the necessity of frequent adult boosters, combined with data supporting coadministration and catch-up flexibility, suggests that context-specific adaptation of guidelines may provide optimal protection. In disaster and emergency medicine, ensuring timely wound prophylaxis, verifying immunization status, and incorporating women of reproductive age into outreach campaigns are essential components of effective tetanus prevention.

## 5. Social and Geographic Inequalities in Tetanus Vaccine Coverage, Globally and Within the United States

As of 2024, global childhood vaccination coverage remains high but uneven. An estimated 89% of the world’s population had received the first dose of the diphtheria-tetanus-pertussis (DTP1) vaccine, and 85% had received the third dose (DTP3) [9,48]. A total of 111 countries had reached ≥90% coverage with DTP3 in 2024, a decline from 125 countries in 2019 [49]. In the U.S., coverage remains high with 98% coverage for DTP1 and 94% coverage for DTP3 in 2024 [49]. Despite these gains, approximately 20 million children worldwide remain unvaccinated or undervaccinated with fewer than three DTP doses [49].

Global estimates of adolescent and adult tetanus booster uptake are more difficult to ascertain due to variable reporting and differing national recommendations. In the U.S., the CDC’s 2024 National Immunization Survey found that 91.3% of adolescents aged 13–17 years had received the recommended Tdap booster dose [50]. Among U.S. adults, 64.2% of individuals aged ≥ 18 years reported ever receiving a Td or Tdap booster [51], and only 59.2% of adults aged ≥ 19 years had received a tetanus booster within the past decade as of 2022 [52].

There are many factors that influence disparities in tetanus vaccination coverage both within the U.S. and globally. Income, educational attainment, race and ethnicity, and broader social contributors of health significantly shape access to TTCV, resulting in uneven protection across populations.

Household income in the U.S. has a well-documented association with tetanus vaccination coverage. A 2015 study analyzing responses to the 2013 Behavioral Risk Factor Surveillance System (BRFSS) found that household income above $50,000 was independently associated with greater Td coverage over the past eight years, and household income over $75,000 was independently associated with greater Tdap coverage over the same period [18]. Data from 2015 to 2017 in the BRFSS supported this, finding that a household income over $50,000 was associated with greater Tdap coverage compared to a household income below $25,000 [19]. The CDC’s Child National Immunization Survey found that, among children born between 2016 and 2020, those living below the federal poverty level had significantly lower coverage with four or more Tdap doses by 24 months of age compared to those at or above the federal poverty level [20]. Household income also has a global effect on tetanus vaccination uptake. A study analyzing DTP3 coverage by wealth quintiles among 51 LMICfound a statistically significant increase in coverage in the highest quintile (wealthiest households) compared to the lowest quintile (poorest households) in 29 countries by using a ratio of the vaccination rates [24]. Furthermore, 20 countries showed a difference of 20+ percentage points for DTP3 immunization in favor of the highest quintile when compared to the lowest [24]. Additionally, 20 countries showed a difference of 20+ percentage points for DTP3 immunization in favor of the highest quintile when compared to the lowest [24].

Educational attainment similarly influences adult tetanus vaccination rates in the U.S. Analysis of the 2016 BRFSS showed that adults with at least one year of college education had significantly higher odds of being up-to-date with receipt of Tdap or Td vaccination compared with high school graduates [21], while not completing high school was associated with lower odds of having coverage compared to high school graduates [21]. Findings from the 2013 BRFSS revealed a similar pattern, with college graduates and those having at least some college education demonstrating significantly higher tetanus booster coverage; however, this earlier analysis did not detect a significant difference between high school graduates and non-high school graduates [18]. Data from the 2015–2017 BRFSS further supported the positive association between higher education and Tdap uptake [19]. Among foreign-born adults in the US, those with an associate’s degree or higher had substantially greater Td and Tdap coverage than those without a high school diploma [22].

Race and ethnicity have also been associated with tetanus vaccination rates in the United States. The 2016 BRFSS demonstrated that Black (non-Hispanic) and Hispanic adults had significantly lower odds of being up-to-date with Td and Tdap compared with White (non-Hispanic) adults [21]. This is consistent with results from the 2013 BRFSS, which additionally showed that Asian adults (non-Hispanic) had lower tetanus booster coverage compared to White (non-Hispanic) adults, whereas American Indians/Alaskan Native (non-Hispanic) adults had higher rates of vaccination against tetanus [18]. Among children in the U.S. born between 2016 and 2020, lower coverage with four or more doses of DTaP by 24 months of age was found for Blacks (non-Hispanic), Hispanics, and American Indians/Alaskan Natives (non-Hispanic) compared to Whites (non-Hispanic), although this difference was not statistically significant after adjustment [20]. For U.S. adults aged ≥ 19 years, racial and ethnic disparities in tetanus vaccination remain substantial. Data from the 2018 National Health Interview Survey (NHIS) show that Black, Hispanic, and Asian adults had significantly lower rates of Td and Tdap receipt in the preceding 10 years compared with White adults, with the exception of Asian healthcare personnel [14]. Similar disparities in Td coverage were identified in the 2012 NHIS, although those findings were not stratified by healthcare occupation [15]. More detailed analyses of NHIS data from 2015 to 2018 by Wang et al. revealed heterogeneity among Asian subgroups: Chinese respondents had significantly lower Td/Tdap coverage compared to White (non-Hispanic) adults, whereas Filipino, Asian Indian, and Other Asian respondents did not differ significantly [16].

Tetanus vaccination is also affected by birthplace. Those aged 19 and over who were born in the U.S. had higher Td and Tdap coverage than those who were born abroad, according to data from the 2018 NHIS [14]. This pattern was also seen in the 2012 NHIS, which found that, among foreign-born adults, Tdap coverage from 2005 to 2012 was considerably higher for those who had lived in the US for less than 10 years compared with ≥10 years, and Td and Tdap coverage was higher among those who were U.S. citizens compared to those who were not [17]. Additionally, among the foreign-born, having been to a doctor in the past year, being younger age, and having an associate’s degree or higher were all associated with greater Td and Tdap vaccination rates [22]. Within all Asian subgroups (Chinese, Filipino, Asian Indian, and Other Asian), those who were born in the U.S. had significantly greater Td and Tdap coverage than those who were born outside of the U.S. [16]. A 2023 study found that the prevalence of sero-immunity against tetanus was lower for foreign-born individuals in the U.S. compared to those born in the U.S. [53].

There are also regional differences in tetanus vaccination rates both within the U.S. and globally. According to data from the 2022 BRFSS, coverage with Td or Tdap for adults aged 18 or over was highest in Vermont, with a rate of 78.7%. The coverage was lowest in Mississippi (50.3%). In 2019, county-level coverage with Td or Tdap was highest in Scott County, MN (86.8%) and lowest in Holmes County, MS (44.7%) [51]. As of 2024, according to United Nations Children’s Fund (UNICEF), the regions of Europe and Central Asia (ECAR), and South Asia (ROSA) had the highest DTP3 coverage at 92%. DTP3 receipt is used by WHO to mark completion of their recommended childhood vaccination series for tetanus. The region of West and Central Africa (WCAR) had the lowest DTP3 coverage at 72% [49]. DTP1-used as a proxy for access to vaccination services-shows a similar pattern, with 2024 coverage ranging from 97% in non-program countries (higher-income nations not targeted by specific WHO immunization support programs) to 81% in WCAR. Nigeria (15%), India (6%), and Sudan (6%) account for the largest share of children who have never received a single DTP dose [49], and Sudan currently has the lowest DTP1 (48%) and DTP3 (39%) coverage globally [48].

Within the US, geographic context plays a significant role in vaccination uptake. Adolescents living in rural counties were less likely to have made a vaccination visit (for any vaccine) than those in urban counties and adolescents living in rural areas are disproportionately represented among youth who have not received any of the recommended adolescent vaccines such as HPV, Tdap, or meningococcal conjugate against *Neisseria meningiditis* serogroups A, C, WY (MenACWY) vaccines [48,50].

Other factors associated with adult Td and Tdap coverage in the U.S. include age, health insurance status, sex, and number of physician visits. Younger adults consistently demonstrate higher tetanus vaccination rates than older adults [14,15,17,21,53]. Being female is associated with lower odds of up-to-date Td vaccination, although Tdap vaccination was noted to be significantly higher among females compared to males in one study analyzing the 2013 BRFSS [15,18,21]. Having health insurance is also associated with higher rates of protection against tetanus for adults [14,15,18,21]. Similar disparities are observed in childhood immunization: Data from the CDC show that children born in 2019–2020 who were insured, and especially those with private insurance, had significantly higher coverage with ≥3 DTaP doses by age 24 months compared with uninsured children or those covered by Medicaid [20]. Healthcare utilization also matters—adults who reported at least one physician visit in the past year had markedly higher tetanus vaccination rates [14,15,18]. Many of these same factors influence tetanus vaccination among foreign-born adults. In this population, younger age, having private health insurance, and having visited a physician in the past year are each independently associated with higher Td/Tdap coverage [22].

Social, demographic, and regional factors play a key role in MNTE, shaping maternal vaccination rates and impacting the likelihood of infant protection at birth. In the U.S., gaps in maternal Medicaid coverage contribute to significant disparities: In states without full Medicaid coverage for pregnant women, Tdap uptake among pregnant women was 20% lower in rural areas than in urban areas [23]. Uninsured pregnant women in rural areas had even lower coverage-27% less than uninsured pregnant women in urban areas [23]. Similar challenges are seen globally. In a study of 60 LMIC, 33.1% of pregnant women had not received a TTCV before delivery, and only 32.8% of pregnant women had received two or more doses by the time of delivery [54]. Factors positively associated with higher maternal TTCV uptake include higher educational attainment, employment, health insurance coverage and having at least one antenatal care visit [54]. Consistent with these findings, Yusuf et al. found that children from wealthier households had significantly greater odds of being protected against tetanus at birth [55].

Despite these challenges, global MNTE progress since 2000 has been substantial. As of December 2024 only 10 countries had not yet achieved MNTE [10]. This contrasts sharply with statistics from the year 2000, at which time 57 countries had not achieved MNTE [10]. From 2000 to 2022, 47 out of 59 priority countries achieved MNTE, as Timor-Leste and South Sudan became priority countries after their independence. In 2022, 16 priority countries had antenatal coverage of ≥80% with at least two TTCV doses, and 30 of 41 priority countries with available data showed improved maternal tetanus vaccination coverage since 2000. Globally, from 2000 to 2021, neonatal tetanus cases decreased by 89% and neonatal tetanus deaths decreased by 84%. Additionally, there was a 12% increase in the proportion of infants protected against tetanus at birth. Unfortunately, the COVID-19 pandemic hindered MNTE progress. Concerningly, since 2020, there were reported increases in neonatal tetanus cases in 18 out of the 59 priority countries [56]. Many of the countries who have not yet achieved MNTE are in the WHO African region, which accounts for approximately 90% of global neonatal tetanus cases [8]. As of 2024, there were 3512 reported cases in this region [48]. However, from 2000 to 2015, regional coverage with at least two TTCV doses increased from 62% to 77%, accompanied by a 75% decrease in reported neonatal tetanus cases [56]. In the countries yet to achieve MNTE, persistent barriers identified include long travel distance to healthcare facilities, transportation and cost constraints, limited antenatal care access and broader socioeconomic inequities [55].

These extensive inequalities and structural barriers to protection against all forms of tetanus highlight critical issues globally. It is imperative that these disparities be addressed, especially in areas that are more susceptible to natural disasters, where disruptions in healthcare services, increased injury risk, and existing inequities can compound vulnerability to tetanus and hinder MNTE progress.

## 6. Impact of Natural Disaster

Natural disasters have long posed serious public health challenges, but their impact has created a greater sense of urgency with climate change, creating the potential for more frequent and more severe disasters. Flooding has recently become the most common natural disaster globally [57]. The number of people affected by coastal and river flooding is expected to reach 147 million by the year 2030 [58]. With natural disasters comes an increased risk of disease outbreak, due to factors such as overcrowding, population displacement, and in the case of tetanus-traumatic injuries from debris and environmental contamination [25].

Tsunamis, and the flooding they generate, have been associated with tetanus outbreaks. Flooding can mobilize *C. tetani* spores from the soil, increasing exposure risk [26]. In the aftermath of the 2004 Indian Ocean earthquake and tsunami, which heavily impacted Aceh Province in Indonesia, 106 tetanus cases were reported within one month—at the time this was the largest number of documented cases after a natural disaster. Twenty patients died, yielding a case fatality rate of 18.9%. Most infections were believed to result either from injuries sustained during the tsunami or from wound contamination acquired while searching through debris in the aftermath [27,28].

After Japan’s earthquake and tsunami in 2011, nine tetanus cases were recorded, none of which resulted in death. Among the seven cases examined in one study, all patients had sustained wounds and four required mechanical ventilation. Notably, none of the patients had reliable vaccination records to confirm tetanus immunity [29]. Japan has been identified as a country at elevated risk for tetanus outbreaks following a tsunami or floods [26], a vulnerability reflected in its ranking as the 4th most at-risk country in the 2021 Global Climate Risk Index [59]. However, the most up-to-date ranking places them at 34th [60]. Regardless, this report and the outbreak after the 2011 tsunami highlight the possibility of tetanus outbreaks after natural disasters even in more developed countries, as climate change accelerates the frequency and severity of extreme weather events.

Earthquakes without concurrent tsunamis have also led to tetanus outbreaks on numerous occasions. After the 2006 earthquake in Yogyakarta, Indonesia, 71 individuals were reported to have tetanus, of which 26 died [28,30]. Twenty-six of the 71 cases were analyzed further, and it was found that eight of those patients died. Of these 26 cases, being located 15 or more kilometers from the hospital was significantly associated with an increased risk of mortality [28,30].

Following the 2005 Kashmir earthquake, one of the largest documented post-disaster tetanus clusters occurred in Pakistan. Within 30 days of the 7.6-magnitude earthquake, 139 tetanus cases and 41 deaths were reported, reflecting a case-fatality rate of approximately 29% in a population with low baseline immunization coverage [7]. The surge in cases was attributed to crush injuries, delayed access to care, and widespread contamination of wounds with soil and debris. The cluster primarily affected younger survivors who lived long enough after the earthquake to present with wound infections, a pattern characteristic of post-disaster tetanus outbreaks. These data highlight how tetanus can rapidly emerge in disaster settings lacking robust vaccination programs and underscore the need for immediate wound care, TTCV administration, and TIG availability during emergency response operations.

Likewise, following the 2010 Haiti earthquake—considered the deadliest natural disaster in the history of the Western Hemisphere—the United States Naval Ship (USNS) *Comfort* deployed as part of Operation Unified Response and served as a major tertiary-care referral center for patients with complex traumatic injuries. In this setting of soil-contaminated crush injuries, open wounds, delayed access to medical care, and widespread disruption of the public health infrastructure, tetanus emerged as a predictable clinical threat. Six patients with tetanus were treated aboard the USNS *Comfort*, five of whom required assisted ventilation [31], reflecting the severity of disease in an under-immunized population [32,33]. Two of the patients died—one was a baby with tetanus [61] and the other an elderly man [31]—highlighting the profound vulnerability of individuals at the extremes of age to severe tetanus outcomes.

Early limitations in vaccine supply initially restricted prophylaxis to the most severely injured patients; however, once supplies stabilized, all patients with wounds received tetanus toxoid and human TIG in accordance with clinical best practices. Additional documentation described extensive wound contamination and polymicrobial infections, creating biologic conditions highly conducive to *C. tetani* germination [62].

Importantly, the senior author (ME) of the present review also served as the U.S. military neurologist on board the USNS *Comfort* during this mission, providing direct clinical management of the tetanus cases and firsthand insight into the challenges of delivering neurological and critical care in a complex humanitarian emergency [32,33].

These six cases represent the experience of a single tertiary-care platform and therefore do not capture the full extent of tetanus following the 2010 Haiti earthquake. Other medical organizations responding to the disaster also reported tetanus cases, underscoring the widespread risk across affected regions. For example, Médecins Sans Frontières documented six additional cases treated in their facilities [63]. Together, these reports demonstrate how quickly tetanus can emerge after large-scale disasters and reinforce the critical importance of timely wound prophylaxis, adequate TTCV and TIG supply, and coordinated clinical preparedness during humanitarian operations. They also highlight how profoundly under-immunization can increase a community’s vulnerability in the aftermath of a catastrophic event.

In the US, where tetanus coverage is generally high, there were no recorded tetanus outbreaks after major recent natural disasters. In the aftermath of Hurricane Katrina in 2005, there were no spikes in recorded infectious disease cases in three affected counties in Mississippi. Many people in this area, however, did visit healthcare facilities to receive the tetanus vaccine after the hurricane, despite CDC recommendation that this is not necessary if a person is up-to-date on their vaccinations [64]. A CDC report encompassing multiple states affected by Hurricane Katrina did find an increase in cases of dermatologic, respiratory, and diarrheal conditions among evacuees and rescue personnel, but tetanus was not mentioned [65]. Investigation of the incidence of reportable diseases in New York City following the devastation of Hurricane Sandy in 2012 found a significant increase only in legionellosis, but not in tetanus [66]. Similarly, after Hurricane Harvey in 2017, those impacted by the flooding reported significantly higher rates of skin rashes, headaches, runny nose, and illnesses, but an increase in tetanus cases was not specifically identified [67]. According to the CDC, tetanus cases that arise in the U.S. are often sporadic, and there have been under 40 reported cases per year since 2010. Those who are most at risk of tetanus are either unvaccinated or undervaccinated [13].

In natural disaster settings, when tetanus is suspected in patients, a standardized set of clinical interventions is recommended to reduce morbidity and prevent further toxin activity. Tetanus toxoid-containing vaccines and human TIG are administered intramuscularly to provide active and passive immunity, respectively; equine tetanus antitoxin may be used when human TIG is unavailable. These interventions are not necessary if the patient’s immunization status is known to be up to date [7,68], but in disaster contexts—where vaccination records are often incomplete or unavailable—TTCV and TIG are recommended for those with dirty or contaminated wounds [32]. Prompt wound management remains essential: thorough cleaning, aeration and surgical debridement help remove necrotic tissue and eliminate anaerobic environments that support *C. tetani* proliferation. Antibiotics, particularly metronidazole, are also used to suppress *C. tetani* growth and proliferation. Metronidazole is preferred over penicillin because of its higher capability to penetrate tissues and due to the GABA-antagonistic activity of penicillin which could potentiate the symptoms of tetanus and exacerbate spasms. To reduce the effects of tetanus, benzodiazepines are also used to control muscle spasms, as they are GABA agonists [7,32,33]. As utilized in the aftermath of the Japan earthquake and tsunami, magnesium sulfate is another option to prevent spasm [7,29]. Severe cases may require airway protection, mechanical ventilation, or tracheostomy, though feasibility varies depending on the available resources and the operational context [7,29,33].

The recurrent emergence of tetanus outbreaks following natural disasters underscores an ongoing global vulnerability, particularly as climate change increases the frequency and severity of extreme events. Despite global increase in tetanus vaccination over the past decades, many countries at heightened climate risk remain below the global average for DTP1 and DTP3 coverage [60]. Many sociodemographic factors, as identified earlier, can exacerbate the effects of natural disasters and elevate tetanus risk. Ensuring adherence to recommended post-exposure interventions, maintaining adequate TTCV and TIG supplies, and reinforcing vigilance among healthcare workers and responding organizations are critical steps to minimizing mortality and preventing outbreaks in disaster-affected populations.

## 7. Strategies for Prevention and Outbreak Protection

Natural disasters such as hurricanes, floods, and earthquakes significantly increase the risk of tetanus infections due to a surge in traumatic injuries, contaminated wounds, and disruption of healthcare services. Collapsing structures and debris cause crush injuries and open wounds, which are often contaminated with soil, feces, or saliva, creating ideal conditions for *C. tetani* infection [69]. Displacement and damaged infrastructure can interrupt routine vaccination programs and delay wound care, further elevating risk, especially in populations with low baseline immunization coverage [28]. American College of Physicians and CDC guidelines emphasize wound management and vaccination status assessment. Tetanus toxoid-containing vaccines are indicated for wound management if >5 years have elapsed since the last dose, with Tdap preferred for those not previously immunized. Tetanus immune globulin is recommended for contaminated wounds in incompletely vaccinated individuals [36,39]. Optimal disaster response strategies include pre-positioning vaccine and TIG supplies, training emergency responders, integrating tetanus prophylaxis into disaster response algorithms, and establishing surveillance and educational outreach programs. The literature underscores the need for broader, standardized protocols to ensure rapid, equitable vaccine access in humanitarian crises [70]. Direct comparative effectiveness data between mass vaccination and targeted strategies remain limited, but targeted approaches are supported by outbreak containment evidence and operational feasibility. These challenges underscore how disaster-related vulnerabilities, including limited access to care, disrupted vaccine supply chains, and gaps in immunization status verification, mirror many of the systemic barriers present under routine conditions. Even outside of crisis settings, incomplete vaccination and inconsistent booster adherence continue to leave portions of the U.S. population susceptible to tetanus.

Despite clear national guidelines for adult tetanus vaccination, adult vaccination coverage rates in the U.S. remain suboptimal. Rates have been shown to decline with age, and significant gaps are observed among immigrants, refugees, and older adults [71,72]. Notably, only 28% of adult immigrant and refugee patients in a recent U.S. health system analysis had documentation of a complete 3-dose tetanus/diphtheria series, and electronic health record (EHR) systems often fail to identify those overdue for vaccination during medical encounters [73]. Adherence to booster guidelines remains another hurdle. Despite the CDC recommendations that every adult should receive a decennial booster, up-to-date booster rates among adults drop below 50% by age 29 and continue to decrease in older age groups [11,71]. Contributing barriers include lack of awareness among patients and providers, missed opportunities during healthcare encounters, incomplete documentation, and logistical challenges in vaccine stocking and administration [74,75,76].

To address these gaps, several evidence-based strategies are recommended. Provider-focused strategies with the strongest evidence include clinician reminders and standing orders to prompt vaccination during visits [76,77]. Provider recommendation is consistently identified as the most impactful behavioral intervention, with meta-analyses showing it increases vaccine uptake by more than threefold [78]. Routine assessment of vaccination status at every patient encounter and ongoing provider education are essential to improve awareness and ensure guideline-concordant care.

System-level interventions are also critical for overcoming missed opportunities and documentation gaps. Clinical decision support tools and EHR alerts can identify overdue patients and prompt both providers and patients, though their effectiveness varies and is maximized when combined with other strategies [73]. Bundled interventions such as combining reminders, standing orders, and workflow changes yield greater improvements in vaccination rates than single interventions alone [79]. Vaccine hesitancy also presents a challenge. The most effective strategies for targeting tetanus vaccine hesitancy and improving patient education are multicomponent and tailored interventions that address both individual and system-level barriers. Provider-focused interventions are central, as the use of motivational interviewing and evidence-based responses to specific safety or ingredient concerns have been shown to increase vaccine acceptance [80,81]. The American College of Cardiology emphasizes that education delivered by primary care physicians during routine visits is particularly effective, and that addressing common misconceptions, such as the belief that vaccines cause the diseases they prevent, should be a routine part of patient counseling [80].

Pregnant women represent another key demographic with persistently low tetanus immunization rates. A large EHR analysis covering 2017–2021 found that 55.1% of pregnant women received Tdap during pregnancy, with rates declining slightly from 56.6% in 2017 to 52.1% in 2021 [82]. These rates remain below the goal of universal coverage recommended by the American College of Obstetricians and Gynecologists and the CDC, and significant disparities persist by insurance status, race/ethnicity, and geography [83,84]. Patient-level barriers include lack of knowledge about the need for Tdap in every pregnancy, concerns about vaccine safety for themselves and their infants, and misconceptions about vaccine effectiveness [85,86,87]. Provider-level barriers such as limited staff training, incomplete documentation, and financial concerns (including cost and insurance coverage, especially for Medicaid-insured women) further impede uptake [88]. Interventions shown to improve Tdap vaccination rates include multi-component strategies that address both patient and provider barriers. Effective approaches are provider education and reminders, standing orders for vaccination, and ensuring vaccine availability on-site during prenatal visits, which together have demonstrated significant increases in uptake [89,90]. Automated EHR reminders and integration of Tdap into routine antenatal care workflows are particularly impactful, with studies reporting increases in coverage from below 50% to over 90% in some settings [91]. These findings underscore the importance of system-level changes, targeted outreach, and consistent provider engagement to close gaps in maternal Tdap immunization.

Despite these challenges, widespread immunization has made tetanus cases in the U.S. relatively rare. Of the sporadic cases that do still occur, the majority arise in individuals without adequate immunization or those who do not receive appropriate PEP after injury. Recent data show that compliance rates with PEP protocols in emergency and wound-care settings are suboptimal, especially among adults with injury-related exposures. A California review found that among tetanus patients with acute injuries, only 22% of those who sought medical care received appropriate PEP according to guidelines [92]. Similarly, a study of high-risk foot puncture wounds in a U.S. emergency department reported that 20.8% of patients who were not up-to-date on tetanus immunization received no form of tetanus prophylaxis, and only 79.2% received a vaccine booster as recommended [93]. These inconsistencies in compliance have been attributed in part to lack of provider training, difficulty obtaining accurate vaccination records at the time of injury, and limited vaccine availability [36,39,94]. Provider education programs including targeted training on wound management protocols and dissemination of updated guidelines have been shown to improve compliance and vaccine uptake [95]. Therefore, targeted interventions that address provider knowledge gaps, such as continuing education and point-of-care training, may be effective in increasing adherence to recommended PEP protocols and reducing instances of disease outbreak [96].

Robust surveillance of such cases is fundamental to sustaining tetanus control and ensuring rapid outbreak detection. In the U.S., tetanus is monitored through the CDC’s National Notifiable Diseases Surveillance System (NNDSS) and state-level Immunization Information Systems (IIS), which collect and analyze data on vaccination coverage and case reports. Integration of IIS with EHRs allows for real-time identification of under-immunized populations and supports the development and implementation of targeted public health interventions. Timely reporting of suspected cases enables rapid investigation, confirmation, and PEP measures, minimizing the risk of secondary cases [36].

Sustaining tetanus prevention and outbreak protection in the U.S. requires a coordinated and adaptable approach that brings together healthcare providers, public health systems, and communities. Strengthening clinician education, improving EHR-based reminder systems, and expanding vaccination access in prenatal, emergency, and occupational settings can help close persistent coverage gaps. Ongoing investment in robust surveillance infrastructure and consistent adherence to PEP protocols remain essential for maintaining national elimination and ensuring rapid response to sporadic cases. By combining preventive strategies with timely public health action, the U.S. can preserve high levels of population immunity and prevent the reemergence of this entirely preventable disease.

## 8. Limitations

This review has several limitations inherent to its narrative design. As a narrative synthesis rather than a systematic or scoping review, formal inclusion and exclusion criteria, risk-of-bias assessment, and quantitative synthesis were not performed. Although this approach allowed for integration of clinical, epidemiologic, and public health perspectives, it introduces the potential for selection bias and limits reproducibility compared with systematic methodologies.

The review draws on a combination of peer-reviewed literature and gray literature, including public health surveillance reports and guideline documents from organizations such as the CDC and WHO. While inclusion of these sources is necessary to accurately reflect current practice and disaster-response frameworks, such materials may vary in methodological rigor and update frequency. In addition, the focus on English-language sources may have resulted in omission of relevant data from non–English-speaking regions.

Finally, disaster-related tetanus case reports and outbreak descriptions are context-specific and may not be generalizable across settings with differing baseline immunization coverage, healthcare infrastructure, or emergency response capacity. Accordingly, conclusions should be interpreted as a qualitative synthesis intended to inform preparedness and prevention strategies rather than definitive estimates of effect.

## 9. Conclusions

Tetanus remains a preventable but persistent public health threat, driven not by scientific uncertainty but by gaps in vaccination access, adherence, and infrastructure. While widespread immunization has rendered the disease rare in the U.S., disparities in coverage, by geography, socioeconomic status, immigration background, and maternal health, continue to leave certain groups at elevated risk. The global picture reflects similar inequalities, with LMIC still struggling to achieve and sustain maternal and neonatal tetanus elimination despite decades of progress.

In an era of climate instability, natural disasters further expose and amplify these underlying vulnerabilities. Floods, earthquakes, and conflicts disrupt healthcare delivery, compromise vaccine cold chains (the temperature-controlled systems needed to preserve vaccine potency), and create conditions ideal for *C. tetani* infection through contaminated wounds. Strengthening preparedness in disaster-prone areas, through pre-positioned vaccine and TIG supplies, integrated response protocols, and targeted education for healthcare workers, will be critical to preventing outbreak resurgence, both nationally and globally.

Moving forward, tetanus prevention must rely on coordinated, multi-tiered strategies. At the clinical level, reinforcing provider education, improving use of EHR alerts, and implementing standing orders can substantially reduce missed vaccination opportunities. At the systems level, investment in robust surveillance networks, such as the CDC’s (NNDSS) and state (ISS), will allow for rapid detection and response to emerging cases. Addressing vaccine hesitancy through consistent, empathetic communication and culturally tailored outreach is equally vital to sustaining long-term protection. Recent shifts in CDC guidance surrounding childhood immunizations, however, pose an additional risk to tetanus control by potentially weakening the clarity of national vaccine messaging and creating confusion among patients, parents and providers. Ensuring that any policy updates are accompanied by strong, evidence-based communication and clear recommendations will be essential to maintaining confidence in the childhood vaccine schedule and preventing erosion of tetanus immunity at the population level.

Ultimately, eliminating residual tetanus morbidity in the U.S., and sustaining global progress, depends on bridging the gap between existing recommendations and real-world implementation. Equitable access, consistent provider engagement, and resilient public health systems remain the pillars of sustained tetanus control. With strategic coordination and continued vigilance, tetanus can remain a vanishing disease, even in a world facing new public health challenges.

## Figures and Tables

**Table 1:** Summary of CDC and ACIP Recommendations for Tetanus Vaccination and Prophylaxis in the United States (as of November 2025).

Population Group	Recommended Vaccine	Schedule/Interval	Special Considerations
Infants and Children (<7 years)	DTaP (diphtheria, tetanus, acellular pertussis)	5-dose primary series at 2, 4, 6, 15–18 months, and 4–6 years	Use DTaP exclusively for children under 7 years of age. Minimum interval between early doses: 4 weeks
Adolescents (11–18 years)	Tdap (tetanus, diphtheria, acellular pertussis)	1 dose at 11–12 years	Tdap may be administered regardless of interval since last Td. Ensures ongoing protection against pertussis
Adults (≥19 years)	Td or Tdap	Booster every 10 years following completion of primary series	Tdap should replace one Td booster in adulthood if not previously received. Td and Tdap are interchangeable for decennial boosters
Pregnant Women	Tdap	Once during each pregnancy (preferably between 27 and 36 weeks gestation)	Provides passive antibody protection to newborns; recommended regardless of prior immunization status
Wound Management (Post-Exposure Prophylaxis)	Td or Tdap ± TIG (tetanus immune globulin)	Based on vaccination history and wound type	Give Tdap or Td if >5 years since last dose. TIG indicated for contaminated wounds in incompletely immunized or unknown-status individuals
Unvaccinated or Incompletely Vaccinated Adults	Tdap → Td or Tdap → Td	3-dose primary series: initial Tdap, then Td or Tdap at 1–2 months and 6–12 months	Begin with Tdap regardless of interval since prior Td. Follow with boosters every 10 years
Healthcare Personnel	Tdap	Single dose if not previously received; then Td/Tdap every 10 years	Ensures occupational protection and prevents nosocomial transmission

Recommendations summarized from CDC and Advisory Committee on Immunization Practices (ACIP) guidelines [12,36,39,40,41]. Abbreviations: DTaP = diphtheria, tetanus, acellular pertussis; Tdap = tetanus, diphtheria, acellular pertussis (reduced-dose formulation); Td = tetanus, diphtheria; TIG = tetanus immune globulin; CDC = Centers for Disease Control and Prevention; ACIP = Advisory Committee on Immunization Practices.

## Data Availability

No new data were created or analyzed in this study.

## Abbreviations

*C. tetani*	*Clostridium tetani*
MNTE	maternal and neonatal tetanus elimination
DTaP	diphtheria-tetanus-acellular pertussis
LMIC	low- and middle-income countries
U.S.	United States
TeNT	tetanus toxin
HC	heavy chain
LC	light chain
GABA	γ-aminobutyric acid
VAMP	vesicle-associated membrane protein
SNARE	soluble N-ethylmaleimide-sensitive factor attachment protein receptor
SNAP-25	synaptosomal-associated protein, 25 kDa
CDC	Centers for Disease Control and Prevention
ACIP	Advisory Committee on Immunization Practices
WHO	World Health Organization
Tdap	tetanus-diphtheria-acellular pertussis
Td	tetanus-diphtheria
TTCV	tetanus toxoid containing vaccines
TIG	tetanus immune globulin
HPV	human papillomavirus
COVID-19	coronavirus disease of 2019
DTP1	first dose of diphtheria-tetanus-pertussis
DTP3	third dose of diphtheria-tetanus-pertussis
DTP	diphtheria-tetanus-pertussis
BRFSS	Behavioral Risk Factor Surveillance System
NHIS	National Health Interview Survey
UNICEF	United Nations Children’s Fund
ECAR	Europe and Central Asia
ROSA	South Asia
WCAR	West and Central Africa
EAPR	East Asia and Pacific
LACR	Latin America and the Caribbean
ESAR	Eastern and Southern Africa
MENA	Middle East and North Africa
MenACWY	meningococcal conjugate against *Neisseria meningitidis* serogroups A, C, W, Y
USNS	United States Naval Ship
EHR	electronic health record
PEP	post-exposure prophylaxis
NNDSS	National Notifiable Diseases Surveillance System
IIS	Immunization Information Systems
